# Promoting best practice in nucleotide sequence data sharing

**DOI:** 10.1038/s41597-020-0471-1

**Published:** 2020-05-20

## Abstract

Today, *Scientific Data* is refining its standards for new submissions describing nucleic acid sequence data.

We begin by reaffirming our support for the repositories of the International Nucleotide Sequence Database Collaboration^1^ (INSDC, http://www.insdc.org/). *Nature* has required that its authors submit sequence data to a public repository since 1996 (ref. ^2^), and has been a strong supporter of the INSDC. This now forms a central part of the data sharing policies of all Nature Research journals, including *Scientific Data* (https://go.nature.com/2M3FT3z). The interconnected data repositories of the INSDC currently host more than 14 petabases of sequence data, safeguarding our world’s genetic heritage and providing a shining example of effective and fair international cooperation, in an era when such can feel all too rare and all the more necessary in the face of global challenges like the COVID-19 pandemic.

Authors are required to deposit new non-human sequencing data to an INSDC repository prior to submission, even if the data are already in another open repository. Sample metadata should be deposited alongside sequence data to one of the INSDC Biosample databases^3,4^. We regard sequence data published at *Scientific Data* and shared through the INSDC repositories as being available for unrestricted use by all researchers in a manner that aligns with principles of open science (see ref. ^5^ for discussion of the complexities around this issue). *Scientific Data*, of course, does not ask that authors deposit sensitive human genetic data that require special ethical or privacy controls to these open repositories. Our list of recommended repositories includes options that are suitable for hosting and sharing sensitive human data (http://go.nature.com/2eLHBFP).

We encourage our authors to consider whether they have other data types, like phenotypic or biochemical data, or processed data outputs, like genomic annotations, that should be included with their submission. *Scientific Data* requires that authors deposit and share all data underlying studies submitted to the journal.

For studies presenting metagenomic or transcriptomic sequencing data, we will now ask authors to declare whether they used any sequencing controls, including negative controls or positive spike-in controls (See e.g.^6–8^). For experimental transcriptomic or epigenomic studies, submissions will be expected to include at least two biological replicates, and to clearly describe the origin of replicate samples. For single-cell sequencing studies, authors should show the results of different normalisation and batch correction methods, whenever feasible.

Lastly, going forward, submissions describing the genome or transcriptome of a single species will generally be declined. With projects like Genome 10K^9^ (https://genome10k.soe.ucsc.edu/) aiming to release thousands of new genomes assemblies, and with metagenomic sequencing routinely generating thousands of microbial assemblies from single studies, it is clear that peer-reviewing and publishing each new assembly independently will not be feasible. We invite groups interested in submitting descriptions of assemblies to the journal to contact us beforehand for advice. We may ask authors to merge papers describing genomes generated with common methods or as part of larger projects.

We feel that these modest refinements of our policies will help the journal continue to meet its aim of publishing datasets of high technical quality and broad reuse value.

## References

[1] Karsch-Mizrachi I, Takagi T, Cochrane G (2017). The international nucleotide sequence database collaboration. Nucleic Acids Res..

[2] Reinforcing access to research data. *Nature* **379**, 191–191, 10.1038/379191a0 (1996). 10.1038/379191a0 8538774

[3] Barrett T (2011). BioProject and BioSample databases at NCBI: facilitating capture and organization of metadata. Nucleic Acids Res..

[4] Gostev M (2011). The BioSample database (BioSD) at the european bioinformatics institute. Nucleic Acids Res..

[5] Amann RI (2019). Toward unrestricted use of public genomic data. Science.

[6] Conesa A (2016). A survey of best practices for RNA-seq data analysis. Genome Biol..

[7] Kim D (2017). Optimizing methods and dodging pitfalls in microbiome research. Microbiome.

[8] Hardwick SA (2018). Synthetic microbe communities provide internal reference standards for metagenome sequencing and analysis. Nat. Commun..

[9] Koepfli K-P, Paten B, and SJO (2015). The genome 10k project: A way forward. Annu. Rev. Animal Biosci..

